# In Vitro and In Silico Study on the Molecular Encapsulation of α-Tocopherol in a Large-Ring Cyclodextrin

**DOI:** 10.3390/ijms24054425

**Published:** 2023-02-23

**Authors:** Mattanun Sangkhawasi, Khanittha Kerdpol, Abbas Ismail, Bodee Nutho, Chonnikan Hanpiboon, Peter Wolschann, Kuakarun Krusong, Thanyada Rungrotmongkol, Supot Hannongbua

**Affiliations:** 1 Center of Excellence in Computational Chemistry (CECC), Department of Chemistry, Faculty of Science, Chulalongkorn University, Bangkok 10330, Thailand; 2 Center of Excellence in Structural and Computational Biology, Department of Biochemistry, Faculty of Science, Chulalongkorn University, Bangkok 10330, Thailand; 3 Department of Pharmacology, Faculty of Science, Mahidol University, Bangkok 10400, Thailand; 4 Institute of Theoretical Chemistry, University of Vienna, 1090 Vienna, Austria; 5 Program in Bioinformatics and Computational Biology, Graduate School, Chulalongkorn University, Bangkok 10330, Thailand

**Keywords:** α-tocopherol, large-ring cyclodextrins (LR-CDs), inclusion complex, phase solubility, molecular dynamics simulation

## Abstract

α-tocopherol is the physiologically most active form of vitamin E, with numerous biological activities, such as significant antioxidant activity, anticancer capabilities, and anti-aging properties. However, its low water solubility has limited its potential use in the food, cosmetic, and pharmaceutical industries. One possible strategy for addressing this issue is the use of a supramolecular complex with large-ring cyclodextrins (LR-CDs). In this study, the phase solubility of the CD26/α-tocopherol complex was investigated to assess the possible ratios between host and guest in the solution phase. Next, the host–guest association of the CD26/α-tocopherol complex at different ratios of 1:2, 1:4, 1:6, 2:1, 4:1, and 6:1 was studied by all-atom molecular dynamics (MD) simulations. At 1:2 ratio, two α-tocopherol units interact spontaneously with CD26, forming an inclusion complex, as supported by the experimental data. In the 2:1 ratio, a single α-tocopherol unit was encapsulated by two CD26 molecules. In comparison, increasing the number of α-tocopherol or CD26 molecules above two led to self-aggregation and consequently limited the solubility of α-tocopherol. The computational and experimental results indicate that a 1:2 ratio could be the most suitable stoichiometry to use in the CD26/α-tocopherol complex to improve α-tocopherol solubility and stability in inclusion complex formation.

## 1. Introduction

Vitamin E is fat-soluble and can be found in a wide range of natural sources, such as vegetable oils, seeds, almonds, avocados, peanut butter, margarine, fatty fish, and fish liver oil [1]. Various biological actions of vitamin E have appeared to promote human health functions, including high antioxidant activity, suppression of platelet aggregation, augmentation of neurological functions, stimulation of smooth muscle cell development and immunological response, anticancer capabilities, and anti-aging benefits [1,2,3]. Vitamin E mainly includes eight isomers, consisting of α, β, γ, and δ derivatives of tocopherol and tocotrienol [4]. In particular, α-tocopherol ((2R)-2,5,7,8-tetramethyl-2-3,4-dihydro-2H-chromen-6-ol; CAS-number: 10191-41-0) is an important component of vitamin E with high biological activity caused by its efficient absorption and metabolism in the human body [4]. Moreover, α-tocopherol, comprising a chromanol head and a saturated phytyl tail, has the potential to act as a strong antioxidant that is useful to human health. However, its low water solubility and high sensitivity to UV light, oxidation, transition metal ions, and food processing [5] make its application in several fields, such as the food, cosmetic, and pharmaceutical industries, difficult. 

Recent studies have demonstrated the inclusion complexation of α-tocopherol with large-ring cyclodextrins (LR-CDs) as one of the most promising approaches for enhancing the physicochemical properties of α-tocopherol [6,7]. This is because LR-CDs exhibit significantly superior water solubility properties than the commonly used β-CD [8], where LR-CDs are cyclic oligosaccharides consisting of more than eight glucose residues linked by α-1,4-glycosidic bonds [9,10,11]. LR-CDs are not currently commercially available to compare the price with γ-CD. The advantage of LR-CDs is complexation of combined drugs, and they may also have solubility higher or equal to γ-CD [12]. Furthermore, LR-CDs are used in various pharmaceutical applications [13], with several studies reporting their successful development as a vehicle for vitamin E [2,6,7,12,14,15]. 

**Figure 1:**
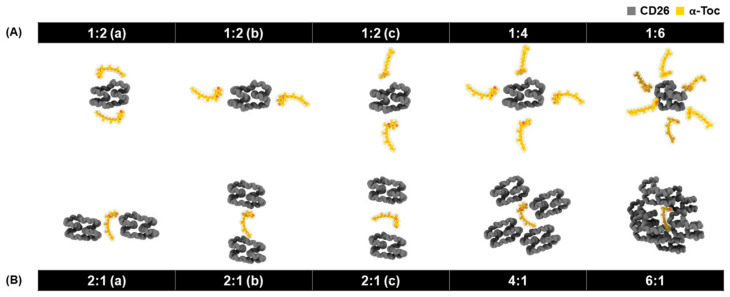
The different initial CD26:α-tocopherol ratios used for the complexes in this study: (**A**) 1:2 (a), 1:2 (b), 1:2 (c), 1:4, and 1:6, and (**B**) 2:1 (a), 2:1 (b), 2:1 (c), 4:1, and 6:1. The complexes were generated by setting the distance between the centers of mass (C_m_) of α-tocopherol and CD26 at 20 Å.

For over a decade, cyclodextrins (CDs) have been investigated through not only experimental but also computational studies. Molecular dynamics (MD) simulations have been extensively applied in the field of CD-based inclusion complexation to understand how CDs entrap a large variety of hydrophobic molecules [16,17,18,19,20,21,22]. In our previous computational study, we investigated the host–guest encapsulation of α-tocopherol with an LR-CD (CD26, containing 26 glucose units) in a 1:1 molar ratio by means of MD simulations [23]. The results showed that CD26 has the ability to effectively encapsulate α-tocopherol, which is likely to increase its aqueous solubility. However, CD26/α-tocopherol inclusion complexation in different molar ratios has not yet been reported and remains open for further investigation. Thus, this study aimed to provide more insight into the complexation between CD26 and α-tocopherol in different molar ratios—1:2, 1:4, 1:6, 2:1, 4:1, and 6:1—at the molecular level (Figure 1). Herein, this phase solubility study of CD26/α-tocopherol complexation was first conducted to determine the possible stoichiometry of the inclusion complex in the solution phase. Subsequently, various inclusion complexes of α-tocopherol with CD26 were generated using computational approaches to investigate their structural and dynamic behaviors in aqueous solution. These investigations are important because the enhancement of the solubility of α-tocopherol by encapsulation into the CDs with suitable molar ratios may improve bioavailability of α-tocopherol. The results will be useful for preparing systems with the most suitable molar ratio in future research. 

## 2. Results and Discussion

### 2.1. Phase Solubility of CD26/α-Tocopherol Complexation 

For the solubility of α-tocopherol at different concentrations of CD26, the following results were obtained: at a CD26 concentration of 0.15 mM, the α-tocopherol concentration rises to 0.22 mM, and at 0.2 mM CD26, a concentration of 0.31 mM is observed. This indicates a 22-fold increase in the concentration of a-tocopherol in comparison to its solubility in pure water (around 0.013 mM). At higher CD26 concentrations, the concentration of the guest molecule decreases significantly. At a CD26 concentration of 0.25 mM and higher, a reduced α-tocopherol concentration of 0.12 mM is found. The determined α-tocopherol concentrations at CD26 concentrations between 0.15 and 0.20 mM are higher than expected for a 1:1 ratio, where according to phase solubility profile of CD26/α-tocopherol complexation (Figure S1), Supplementary Information (SI), the initial linear slope was >1, which indicated higher order of complex formation [24]. Therefore, a higher ratio (for example, a 1:2 ratio) can be postulated. Such a high encapsulation efficiency has been observed in mixtures of LR-CDs (CD9-CD22) complexed with α-tocopherol [7]. In such solutions, the concentrations depend on at least two equilibrium constants and the resulting dependencies on the CD26 concentrations are mostly non-linear. At higher CD26 concentrations, self-aggregation has to be assumed, which leads to a lower solubility of the complexes and therefore a decrease of the α-tocopherol concentration in solution [25]. As a consequence of the experimentally proven complexation with a ratio higher than 1:1, we decided to further vary the ratio of CD26 to α-tocopherol in the complexes as 1:2, 1:4, 1:6, 2:1, 4:1, and 6:1 as the simplest model to investigate their dynamic behavior in aqueous solution using MD simulations. In this way, we explored the most probable configuration in the host–guest encapsulation process at the molecular level, as detailed in the following section.

### 2.2. Stability of the Inclusion Complexes

Root-mean-square deviation (RMSD) calculations were used to detect the system stability of all the studied complexes. The RMSDs of all the α-tocopherol and CD26 atoms in each system were plotted against the simulation time, as illustrated in Figure S2. For all the CD26:α-tocopherol ratios used in the complexes, the RMSD profiles show a similar trend, with RMSD fluctuations around 11–25 Å; the RMSD values of all the systems increase and then fluctuate somewhat until reaching an equilibrium at ~150 ns. One can state that the simulated systems were likely to become stable after an MD simulation time of ~150 ns. Note that 1:2 (c) system is considered as the most stable complex, while the least stable complex system is found in 2:1 (c). Therefore, the trajectories from the last 100 ns of each simulation were adopted as the production phase for further analysis.

### 2.3. Structural Conformation of α-Tocopherol/CD26 Complexes

#### 2.3.1. Distance Analysis and RMSD Clustering 

**Figure 2:**
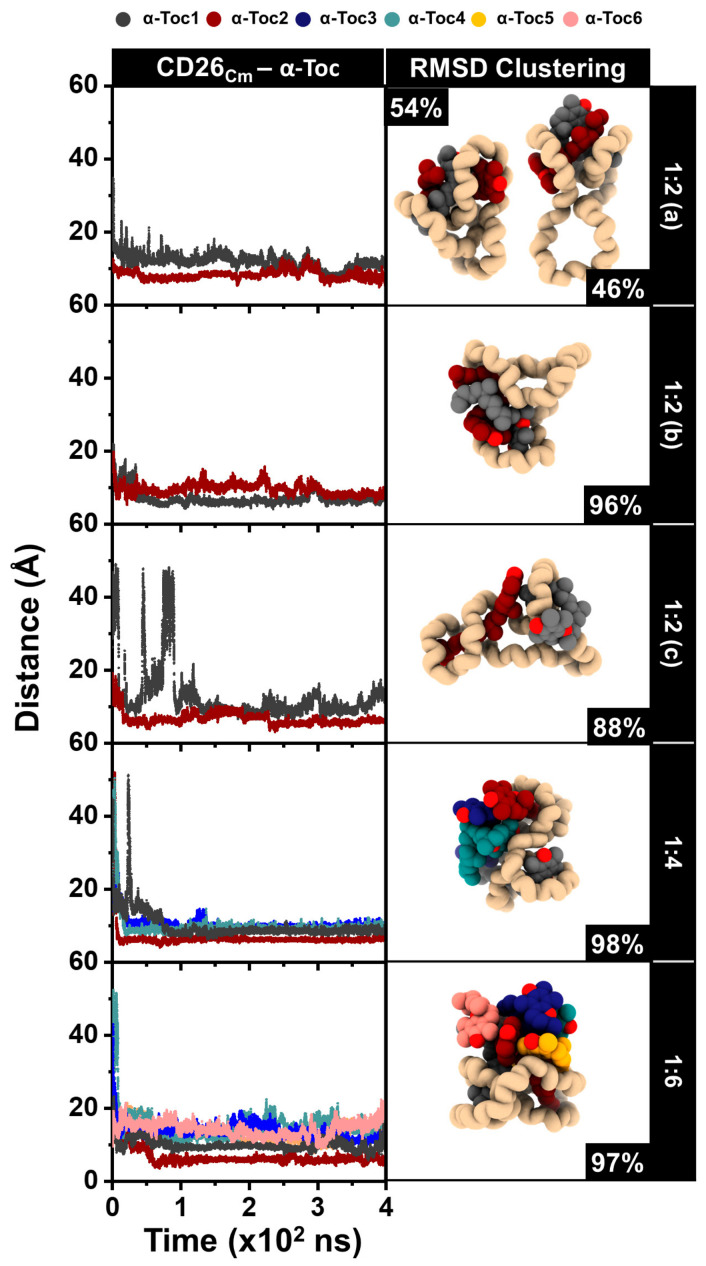
(**Left**) The distances between the C_m_ of CD26 and α-tocopherol and (**right**) RMSD clustering of the 1:2 (**a**), 1:2 (**b**), 1:2 (**c**), 1:4, and 1:6 complexes. The difference between models 1:2 (**a**–**c**) is α-tocopherol’s beginning position arrangement.

**Figure 3:**
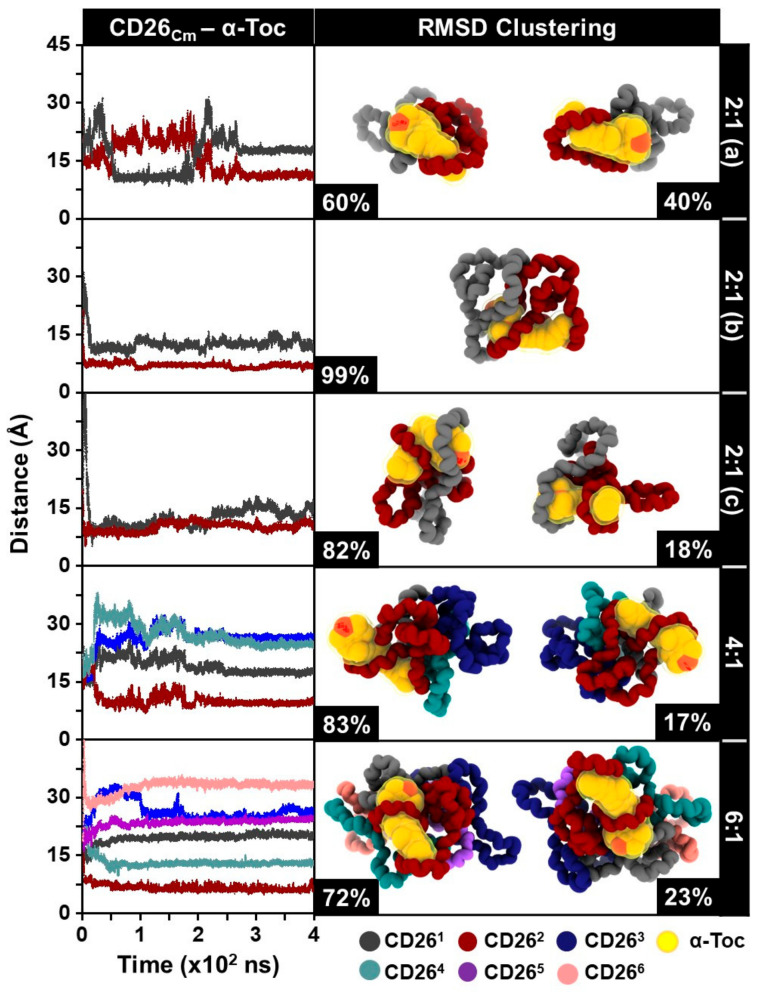
(**Left**) The distances between the C_m_ of CD26 and α-tocopherol and (**right**) RMSD clustering of the 2:1 (**a**), 2:1 (**b**), 2:1 (**c**), 4:1, and 6:1 complexes. The difference between models 2:1 (**a**–**c**) is CD26 beginning position arrangement.

The distance between the centers of mass (C_m_) of CD26 and α-tocopherol in all complexes was plotted against the simulation time, as shown in Figure 2 and Figure 3 (left panel). The representative structures derived from the RMSD clustering for each system were calculated over the last 100 ns of the simulations, as depicted in Figure 2 and Figure 3 (right panel). The RMSD clustering structures in Figure 2 show the folding of the CD26 molecule, which encapsulates both α-tocopherol molecules in the 1:2 (a–c) ratio. In the 1:4 and 1:6 systems, the CD26 molecule can encapsulate only α-tocopherol1 (black) and α-tocopherol2 (red), suggesting that the maximum number of α-tocopherol molecules that CD26 can enclose is limited to two. Likewise, the distance analysis indicates that α-tocopherol1 and α-tocopherol2 are the closest to CD26, at approximately 6–9 Å, in these systems. This implies that upon the addition of more than two α-tocopherol molecules, CD26 can still encapsulate only one to two of these molecules, which agrees well with the phase solubility study proposing a 1:2 CD26:α-tocopherol ratio in the complexes (see Section 2.1) and a previous experimental study of mixed LR-CDs (CD9–CD22) with α-tocopherol [7]. To further explore the encapsulation of α-tocopherol in CD26 at higher concentrations, systems with varying numbers of CD26 molecules were investigated.

Figure 3 highlights the cyclodextrin conformations of CD1 and CD2 in the 2:1 (a), 2:1 (b), and 2:1 (c) systems. The two CD molecules in the 2:1 (a) and 2:1 (b) systems move closer to each other and entrap the α-tocopherol, as can be seen from the distance values of 6–16 Å. Similarly, in the 2:1 (c) system, both CD1 and CD2 are able to tightly encapsulate the α-tocopherol molecule, implying that a 2:1 CD26:α-tocopherol ratio possibly occurs at higher CD26 concentrations. In the case of the 4:1 and 6:1 ratios, CD2 is closest in distance to the α-tocopherol molecule, while the remaining CD26 molecules appear to have aggregated. This strongly supports the phase solubility study, revealing that the self-aggregation of CD26 molecules at high concentrations (>0.200 mM) limits the encapsulation process between CD26 and α-tocopherol. In addition to their different cavity sizes, which may affect the number of guest molecules that bind to each CD molecule, LR-CDs display a variety of mobilities and flexibilities [26], which may lead them to self-assemble at high concentrations, reducing their capacity to form host–guest inclusion complexes [25]. Altogether, our computational results suggest that when the number of CD26 molecules is increased, that is, in the 4:1 and 6:1 ratios, only a single CD26 molecule is able to fully encapsulate α-tocopherol. 

#### 2.3.2. Radius of Gyration and Inclusion Complexes Versus the Simulation Time

**Figure 4:**
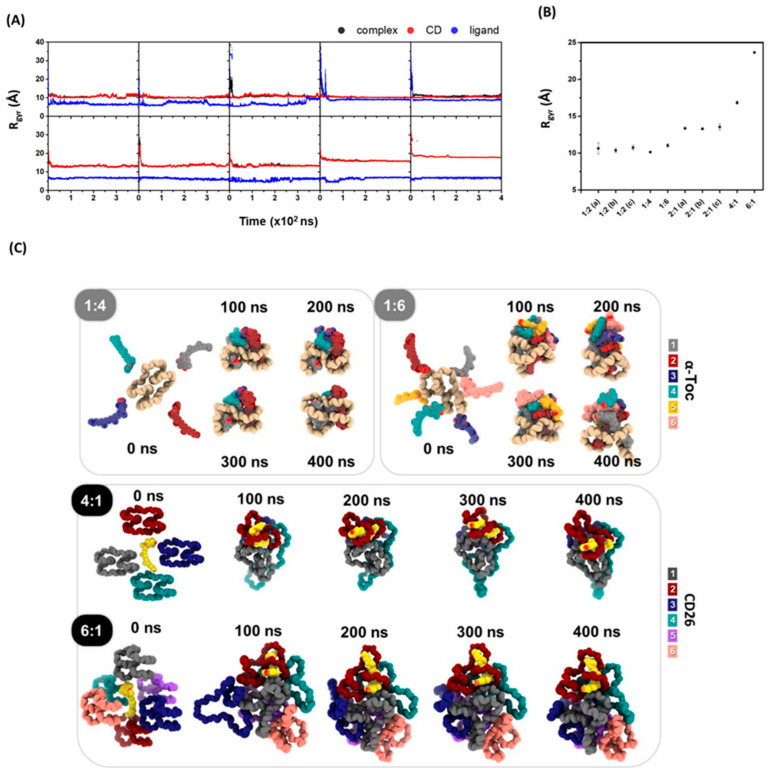
(**A**) The R_g_ of all the CD26/α-tocopherol systems versus the MD simulation time. (**B**) The R_g_ values of the complex atoms averaged over the last 100 ns of the simulations. (**C**) MD snapshots at different time points for the 1:4, 1:6, 4:1, and 6:1 systems.

To measure the conformational flexibility of the overall structures, the radius of gyration (R_g_) was calculated [27]. In each system, the R_g_ values of α-tocopherol, CD26, and the complexes were separately investigated (Figure 4A). The R_g_ profiles of the 1:2 (a–c), 1:4, and 1:6 systems reveal that the complexes have an average R_g_ of approximately 10–11 Å in the last 100 ns of the simulations (Figure 4B), which is notably similar to the case in our previous computational study of the CD26/α-tocopherol inclusion complex at a 1:1 ratio [23]. We also found that increasing the number of α-tocopherol molecules from two to four to six does not significantly affect the overall R_g_ values. On the other hand, the average R_g_ for the 2:1 (a–c) systems is approximately 13 Å, whereas the average R_g_ values are higher in the 4:1 (16.84 Å) and 6:1 (23.65 Å) systems. This is due to the increase in the number of CD26 molecules, which increases the overall size of the system. Moreover, the MD snapshots at different time points (0, 100, 200, 300, and 400 ns) in the 1:4, 1:6, 4:1, and 6:1 systems confirm the self-aggregation of CD26 and α-tocopherol, as depicted in Figure 4C. Our findings indicate that α-tocopherol3 and α-tocopherol4 in the 1:4 system and α-tocopherol3 to α-tocopherol6 in the 1:6 system aggregate at the outer surface of the CD26 molecule. Figure 4C also shows the aggregation of CD3 and CD4 in the 4:1 system and CD3 to CD6 in the 6:1 system. Only CD2 is able to fully encapsulate the α-tocopherol, while CD1 partly attaches to it. One should note that CD self-aggregation is caused by intermolecular hydrogen bonds. The sizes, types, and concentrations of CDs impact their self-aggregation and distribution in aqueous media [24,28].

### 2.4. Atom Contacts and Water Accessibility in Inclusion Complexes

#### 2.4.1. Number of Atom Contacts 

**Figure 5:**
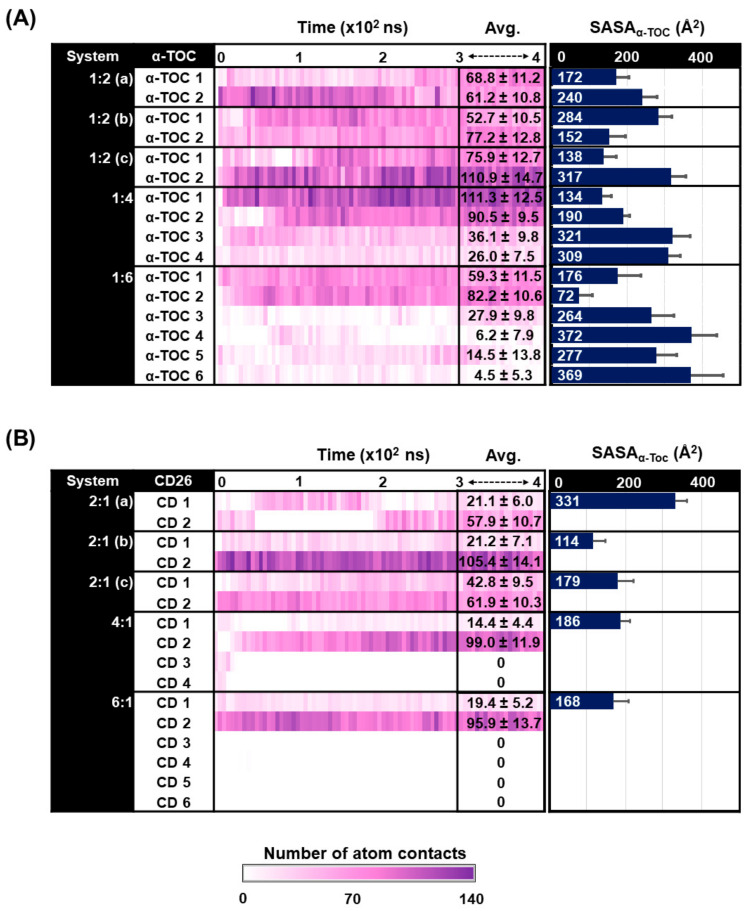
(**A**) The number of atom contacts in the 1:2 (a), 1:2 (b), 1:2 (c), 1:4, and 1:6 complexes (with varying numbers of α-tocopherol molecules) and the SASAs of α-tocopherol in each complex. (**B**) The number of atom contacts in the 2:1 (a), 2:1 (b), 2:1 (c), 4:1, and 6:1 complexes (with varying numbers of CD26 molecules) and the SASAs of α-tocopherol in each complex.

The number of atom contacts between CD26 and α-tocopherol was monitored to evaluate the host–guest encapsulation process. A higher number of atom contacts indicates closer contact between the host (CD26) and guest (α-tocopherol), suggesting that their complexation could occur [23,26,29]. Figure 5 represents the time evolution of the number of atom contacts for each complex, together with its value averaged over the last 100 ns of the MD simulations. The results demonstrate that the number of atom contacts of α-tocopherol1 and α-tocopherol2 in the 1:2 (a–c) systems falls in the range of 60–110, reaching slightly higher values than those in the 1:1 complex (60–90) from our previous study [23]. This reflects that both α-tocopherol molecules were well encapsulated in the cavity of CD26. Likewise, in the case of the 1:4 and 1:6 ratios, α-tocopherol1 and α-tocopherol2 exhibit a larger number of atom contacts than the remaining α-tocopherol molecules, indicating that only one to two α-tocopherol molecules can be encapsulated by a single CD26 molecule. In addition, the results from measuring the number of atom contacts are in good agreement with those of the distance and RMSD clustering analyses, which indicate that the CD26/α-tocopherol inclusion complex was most likely formed in the ratio of 1:2. Similarly, in the 2:1 complex, the number of atom contacts falls in a similar range to that of the systems in which the number of α-tocopherol molecules was varied, as mentioned earlier. For the 4:1 and 6:1 ratios, our findings reveal that CD1 (14.4 ± 4.4 and 19.4 ± 5.2, respectively) and CD2 (99.0 ± 11.9 and 95.9 ± 13.7, respectively) exhibit a higher number of atom contacts than the remaining CD26 molecules. This strongly supports the full encapsulation of α-tocopherol in CD2 and its partial attachment to CD1, as observed in the RMSD clustering analysis (Figure 3) and MD snapshots at different simulation times (Figure 4C), assuming that the inclusion complex of CD26 and α-tocopherol in the 2:1 ratio could also be formed.

#### 2.4.2. Solvent-Accessible Surface Area 

Besides the above-mentioned structural information on the complexes, the solvent-accessible surface area (SASA) was evaluated as compared to the number of atom contacts mentioned in Section 2.4.1. SASA calculations have been commonly applied to explore the impact of water accessibility on CD–guest inclusion complexes [11,17,23,29,30]. In this study, we analyzed the SASAs of all the studied models, focusing on α-tocopherol as the guest molecule. The SASA values of the CD26/α-tocopherol inclusion complexes using α-tocopherol as the atomic radii for the solvent-exposed area are shown in Figure 5. This assumes that the formation of an inclusion complex between α-tocopherol and CD26 decreases the water accessibility surrounding the α-tocopherol molecule. After a simulation time of 300 ns, the SASA value is approximately 138–317 Å^2^ for the 1:2 (a–c) ratio, as shown in Figure 5A. In comparison, the value in our previous study of the 1:1 complex was slightly lower, in the range of 120–303 Å^2^ [23], due to difference in the number of guest molecules. It is also noteworthy that in the 1:4 and 1:6 ratios, the SASA values of α-tocopherol1 and α-tocopherol2 are significantly less than those of the remaining α-tocopherol molecules, indicating that water has less access to these two guest molecules. This also reveals that when the α-tocopherol is encapsulated by CD26, its SASA is lower than when it is located farther from the CD26 molecule.

In Figure 5B, considering the complexes formed by varying the number of CD26 units for a single α-tocopherol molecule, the SASAs are in the range of 114–331 Å^2^, reaching slightly higher values than those in the 1:2 complexes (138–317 Å^2^). Although the 2:1 (a) system exhibits somewhat higher SASA values than the 2:1 (b) and 2:1 (c) ones, this is still reasonable because a lower number of atom contacts was found in this system. 

## 3. Materials and Methods

### 3.1. Solubility Study

Isolation of LR-CDs with a degree of polymerization (DP) of 26 (CD26) was carried out using preparative HPLC, as described previously [26]. The high-performance anion-exchange chromatography with the highly sensitive pulsed amperometric detection (HPAEC-PAD) was used to analyze the isolated HPLC peak fraction containing CD26 before it was lyophilized for 72 h to produce CD26 powder. The purity of isolated CD26 was compared against the cycloamylose standard purchased from Ezaki Glico Co., Ltd. (Osaka, Japan) as shown in Figure S3. A solubility study was performed in a reaction volume of 0.5 mL using the method described by Higuchi and Connors [31]. An excess amount of α-tocopherol (Sigma-Aldrich) was added to 0.5 mL distilled water containing concentrations of CD26 (0 to 0.30 mM). The mixtures were vortexed, ultrasonicated for 15 min, sealed with parafilm, and incubated at 25 °C at 250 rpm, shaking for 72 h. The mixtures were then centrifuged at 10,000× *g* for 15 min to precipitate the insoluble portion. The supernatants were filtered using a 0.22-µm filter syringe, and the absorbance was measured using a BIOTEK microplate reader.

### 3.2. System Preparation 

The original configuration of CD26 was derived from the X-ray crystal structure of cyclomaltohexaicosaose (PDB ID: 1C58) [32]. The 3D structure and partial atomic charges of α-tocopherol and other molecular parameters were taken from our previous study [23]. Furthermore, α-tocopherol and CD26 were treated with the general AMBER force field (GAFF) [33] and the Glycam06j carbohydrate force field [34], respectively. Accelrys Discovery Studio 2.5 (Accelrys Software Inc., San Diego, CA, USA) was used to model the initial structures of α-tocopherol with CD26 in various molar ratios—1:2 (a–c), 1:4, 1:6, 2:1 (a–c), 4:1, and 6:1—with the distances between the C_m_ of α-tocopherol and CD26 set at 20 Å (Figure 1). The simulated systems were then solvated using the Leap module of AMBER16 with a truncated octahedral box and the TIP3P water model [35] with a spacing distance of 10 Å from the complexes. To relax the structure, energy minimization was performed on only the α-tocopherol and CD26 molecules using the steepest descent (SD) method (1000 steps), followed by the conjugate gradient (CG) method (3000 steps). Next, the SD and CG methods were used to minimize the entire system for further MD simulations.

### 3.3. Molecular Dynamics Simulations

MD simulations on the 10 systems under study were performed using the AMBER16 software package [36]. In brief, all starting structures were slowly heated from 10 to 298 K using a canonical ensemble (NVT) for 100 ps and then equilibrated for another 1200 ps. All-atom MD simulations were run in the isothermal–isobaric (NPT) ensemble at 1 atm and 298 K with a simulation time step of 2 fs for a total simulation time of 400 ns. The Berendsen barostat [37], with a pressure relaxation duration of 1 ps, and the Langevin thermostat [38], with a collision frequency of 2 ps^−1^, were utilized to maintain the pressure and temperature, respectively, during the MD simulations. To constrain all chemical bonds involving hydrogen atoms, the SHAKE method [39] was applied, while the particle mesh Ewald (PME) summation approach [40] was used to tackle long-range electrostatic interactions. The cutoff for nonbonded interactions was set to 10 Å. To roughly determine the stability of all systems, the RMSD was monitored. In addition, the distance between the host and guest molecules, RMSD clustering, R_g_, number of atom contacts (#contact), and SASA were analyzed using the CPPTRAJ module of AMBER16.

## 4. Conclusions

α-tocopherol is the most bioactive form of vitamin E and is widely utilized in many fields. Due to its low water solubility, though, LR-CDs have been selected to improve its physicochemical properties. In this study, a solubility study of CD26/α-tocopherol and MD simulations on various complex ratios of CD26 to α-tocopherol (1:2, 1:4, 1:6, 2:1, 4:1, and 6:1) were performed. The solubility results indicate that the host–guest inclusion complex might exist predominantly in the 1:2 ratio. In support of this, all structural findings from the MD simulations suggest that CD26 could effectively serve as a carrier for an optimal number of two molecules of α-tocopherol. Moreover, from the analyses of distance, RMSD clustering, radius of gyration, and number of atom contacts in the models with varying numbers of α-tocopherol molecules, we predict that the most suitable molar ratio of CD26 to α-tocopherol is 1:2. In the complexes with varying numbers of CD26 molecules per one α-tocopherol unit, the results suggest that the most probable ratio is 2:1 due to the CD26 self-aggregation observed in the 4:1 and 6:1 systems, resulting in limited α-tocopherol encapsulation. We therefore propose and recommend that CD/α-tocopherol inclusion complexes be prepared using the 1:2 ratio. The benefits from this study pave the way for the usage of CD26 as alternative pharmaceutical solubilizer with the optimum molar ratio to improve the solubility and probably better bioavailability of α-tocopherol in pharmaceutical applications. Finally, this study serves as a starting model for future research regarding CD/α-tocopherol or other related inclusion complexation.

## Data Availability

All data was include in this manuscript.

## Supplementary Materials

The following supporting information can be downloaded at https://www.mdpi.com/article/10.3390/ijms24054425/s1.
