# Supplementary material for: In Vitro and In Silico Study on the Molecular Encapsulation of α-Tocopherol in a Large-Ring Cyclodextrin
Source: Int J Mol Sci. 2023 Feb 23;24(5):4425. doi: 10.3390/ijms24054425 (PMC10002136; doi:10.3390/ijms24054425)
Supplement: Supplementary file 1 [file ijms-24-04425-s001.zip › ijms-2216520-supplementary.pdf]

Supporting Material

In Vitro and In Silico Study on the Molecular Encapsulation of α-Tocopherol in a Large-Ring Cyclodextrin

Mattanun Sangkhawasi [1,2], Khanittha Kerdpol [1], Abbas Ismail [2], Bodee Nutho [3], Chonnikan Hanpiboon [2], Peter Wolschann [4], Kuakarun Krusong [2], Thanyada Rungrotmongkol [2,5,*] and Supot Hannongbua [1,*]

[1] Center of Excellence in Computational Chemistry (CECC), Department of Chemistry, Faculty of Science, Chulalongkorn University, Bangkok 10330, Thailand
[2] Center of Excellence in Structural and Computational Biology, Department of Biochemistry, Faculty of Science, Chulalongkorn University, Bangkok 10330, Thailand
[3] Department of Pharmacology, Faculty of Science, Mahidol University, Bangkok 10400, Thailand
[4] Institute of Theoretical Chemistry, University of Vienna, Vienna 1090, Austria
[5] Program in Bioinformatics and Computational Biology, Graduate School, Chulalongkorn University, Bangkok 10330, Thailand
* Correspondence: thanyada.r@chula.ac.th (T. Rungrotmongkol), Tel: +66-2218-5426; and supot.h@chula.ac.th (S. Hannongbua), Tel. +66-8163-61957

Keywords: α-Tocopherol; Large-ring cyclodextrins (LR-CDs); Inclusion complex; Phase solubility; Molecular dynamics simulation

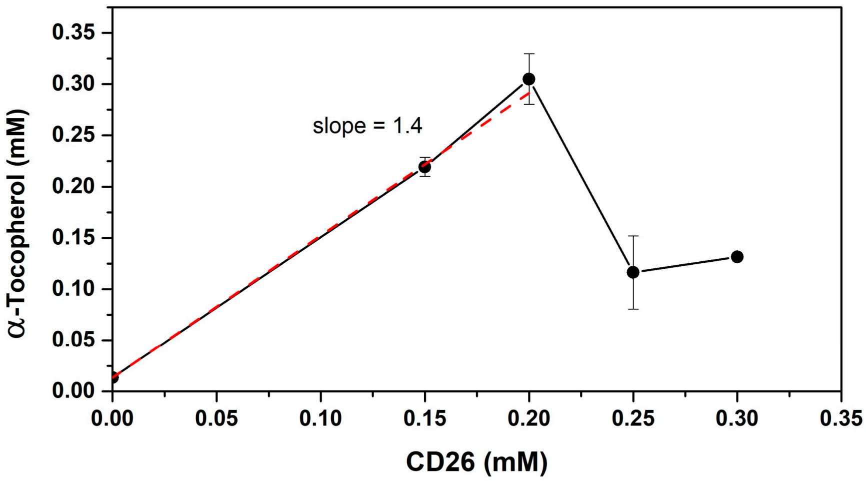

Figure S1. The phase solubility profile of CD26/ a-tocopherol complexation

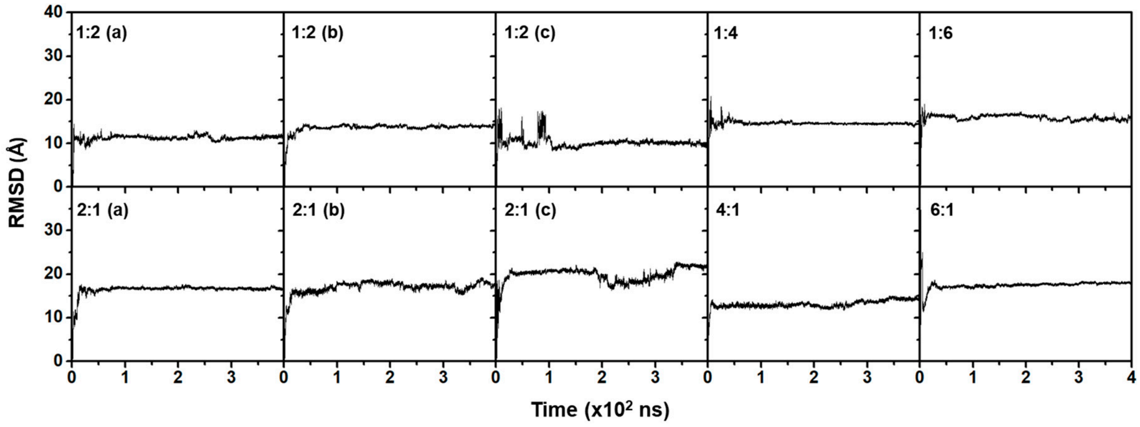

Figure S2. RMSD plots for all atoms of studied complexes along the 400 ns simulation time

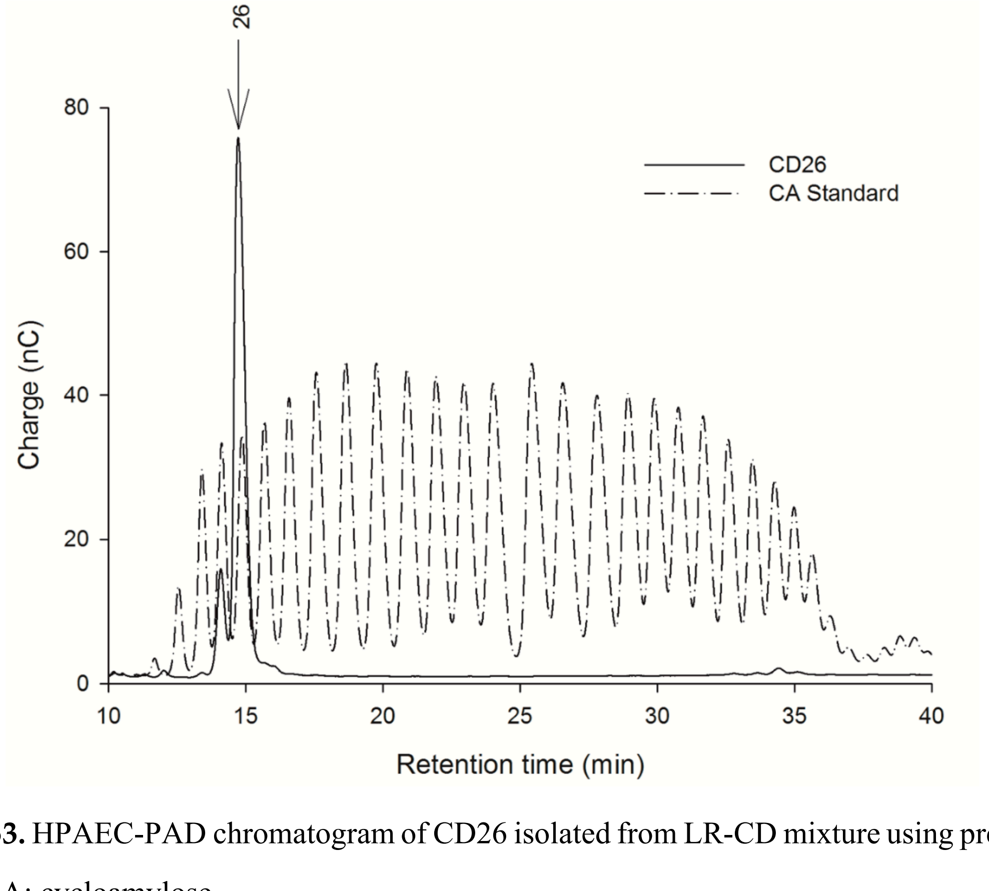

Figure S3. HPAEC-PAD chromatogram of CD26 isolated from LR-CD mixture using preparative HPLC. CA: cycloamylose
